# Translational molecular imaging in exocrine pancreatic cancer

**DOI:** 10.1007/s00259-018-4146-5

**Published:** 2018-09-17

**Authors:** Bart Cornelissen, James C. Knight, Somnath Mukherjee, Laura Evangelista, Catarina Xavier, Federico Caobelli, Silvana Del Vecchio, Latifa Rbah-Vidal, Jacques Barbet, Marion de Jong, Fijs W. B. van Leeuwen

**Affiliations:** 1 CRUK/MRC Oxford Institute for Radiation Oncology, Department of Oncology, Oxford University, Oxford, UK; 2 Istituto Oncologico Veneto I.R.C.C.S., Padova, Italy; 3 Vrije Universiteit Brussel, Brussels, Belgium; 4 Department of Radiology, Universitätsspital Basel, Basel, Switzerland; 5 Universita’ degli Studi di Napoli “Federico II”, Naples, Italy; 6 CRCINA, INSERM, CNRS, Université d’Angers, Université de Nantes, Nantes, France; 7 Department of Radiology & Nuclear Medicine, Erasmus MC, Rotterdam, The Netherlands; 8 Interventional Molecular Imaging Laboratory, Department of Radiology, Leiden University Medical Center, Leiden, The Netherlands

**Keywords:** Pancreatic ductal adenocarcinoma, Molecular imaging, PET, SPECT, Preclinical developments

## Abstract

Effective treatment for pancreatic cancer remains challenging, particularly the treatment of pancreatic ductal adenocarcinoma (PDAC), which makes up more than 95% of all pancreatic cancers. Late diagnosis and failure of chemotherapy and radiotherapy are all too common, and many patients die soon after diagnosis. Here, we make the case for the increased use of molecular imaging in PDAC preclinical research and in patient management.

## Introduction

Effective treatment for pancreatic cancer remains challenging, particularly the treatment of pancreatic ductal adenocarcinoma (PDAC), which makes up more than 95% of all pancreatic cancers. Although research effort has recently been stepped up, the average 5-year survival following the diagnosis of PDAC is a dismal 5% and has not changed at all over the past 40 years. In excess of 50,000 patients were diagnosed with PDAC in the EU in 2012, with nearly as many dying from the disease (source: Cancer Research UK). Radical surgery followed by adjuvant chemotherapy remains the mainstay of curative treatment and is associated with a median overall survival of 28 months, very low compared with that of other cancers. As patients with PDAC often present with vague or nonspecific symptoms, the disease frequently remains undetected until the later stages when invasion of the surrounding vasculature or the presence of metastases prevent radical therapy. In these patients, innate resistance to chemotherapy and radiotherapy are all too common, and many die soon after diagnosis (Cancer Research UK, Pancreatic Cancer UK, and [1]). Nuclear medicine imaging and radionuclide therapy in pancreatic neuroendocrine tumours (P-NETs) have been described in great detail elsewhere [2]. PDAC, originating from the exocrine part of the pancreas is markedly distinct from exocrine PDAC tumours. P-NETs are much more rare, and have a more favourable diagnosis. Here, we make the case for the increased use of nuclear medicine imaging in PDAC preclinical research and in patient management.

Given the late presentation with inoperable disease and poor response to chemo(radio)therapy, there is a clear need for early detection methods, as well as for early and accurate therapy response assessment, so that clinical decisions can be made expeditiously. It is in these aspects that molecular imaging, using techniques such as PET and SPECT, can make a significant difference. The current paradigm for pancreatic cancer care mainly relies on anatomical imaging using ultrasonography, CT and MRI, and on obtaining confirmatory biopsies of the tumour. The disadvantage of these imaging approaches is that differentiation between malignant lesions and nonmalignant cysts is challenging, especially for smaller lesions [3]. Because PET and SPECT imaging can provide functional information about a tumour via its unique biomarker expression, they can be considered complementary to the above techniques and can overcome several of their inherent disadvantages.

Treatment stratification and targeted therapy based on pathological/molecular parameters are revolutionizing the management of many different cancers. Yet, management of pancreatic cancer continues to challenge medical professionals, largely because of the diversity of the pathways that drive this cancer. Moreover, the challenges of obtaining adequate tissue from the tumour itself given its deep-seated position within the body make serial biopsies to assess treatment effects rather impractical. Sampling from a single tumour site ignores the possible extent of tumour heterogeneity and the existence of metastasis with a different genetic and phenotypic make-up. Again, molecular imaging can overcome these limitations, and complement ongoing efforts.

On the whole, biomarkers for pancreatic cancer are underexplored and those few that are in use, such as expression levels of CA19.9 or CEA in blood plasma, are significantly lacking in specificity and sensitivity. Hence, alternative biomarkers are much sought after. A recently published report suggests that a new mixed analyte blood test is more promising [4]. Although this particular test was shown to be able to effectively highlight possible pancreatic malignancy (in approximately 80% of patients, with a specificity of more than 99%), a blood test is unable to reveal either the anatomical location or the extent of the disease. Such a blood test, in conjunction with sensitive noninvasive molecular imaging aimed at detecting early cancerous lesions, could provide a more effective strategy for early diagnosis. Moreover, the use of molecular tracers helps realize imaging-based measures of functional pathways targeted by treatment, and could therefore provide a means to determine treatment response. Several molecular imaging strategies for PDAC are in use in the clinic or are in clinical trials, and several new molecular imaging strategies have been proposed in the literature, but still remain in the preclinical research stage, as described below.

## Clinical molecular imaging of PDAC (PET)

### ^18^F-FDG

**Fig. 1:**
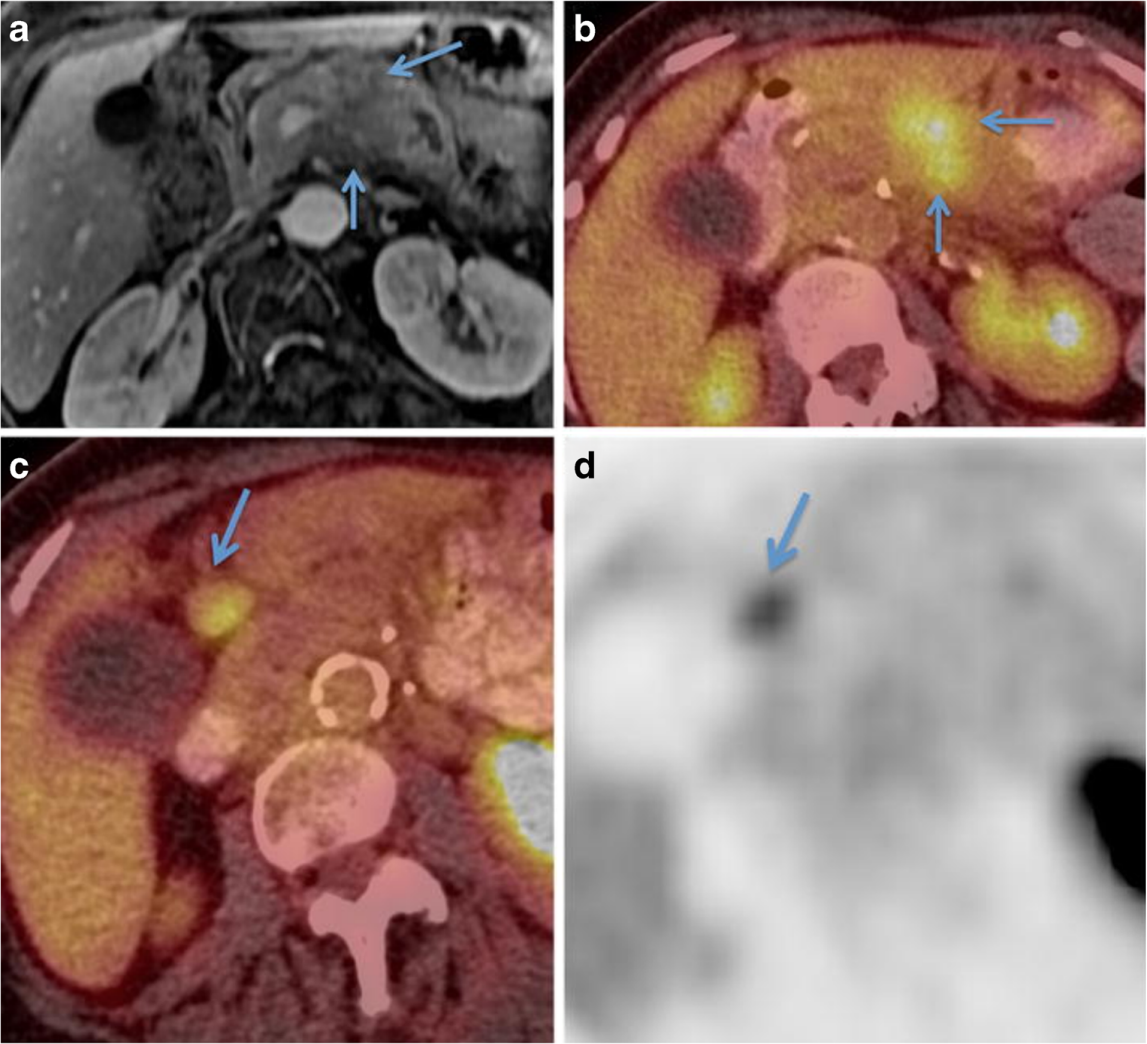
^18^F-FDG PET imaging for initial staging of PDAC in an 80-year-old woman. **a** The initial staging MR image shows a hypoenhancing mass in the pancreatic body (*arrows*) that has resulted in pancreatic ductal dilatation. **b** The PET/CT image shows corresponding FDG uptake in the mass (*arrows*). **c**, **d** Additionally, the fused PET/CT image (**c**) and PET-only image (**d**) show an ^18^F-FDG-avid peripancreatic node (*arrows*). Adapted from Yeh et al. [7]

^18^F-FDG PET imaging, the mainstay of PET imaging, has been considered for a long time to inform the diagnosis of PDAC. Most PDAC tumours have a high metabolic rate and their ^18^F-FDG uptake increases throughout disease progression from pancreatic intraepithelial neoplasia (PanIN) precursor lesions (PanIN-1 to PanIN-3) up to PDAC (the canonical PDAC tumour progression model) [5, 6]. Over 90% of PDAC tumours carry a mutation in the KRAS oncogene, which promotes glucose uptake via upregulation of hexokinase-2 and the glucose transporter [7]. Clinically, ^18^F-FDG uptake in PDAC is considered valuable in treatment selection (Fig. 1). Some guidelines suggest that ^18^F-FDG PET has a very limited or no role in the diagnosis or prognosis of PDAC [8, 9], although there are a number of reports of the superiority of ^18^F-FDG PET/CT in the detection of PDAC over contrast-enhanced CT and MRI [7].

Patients presenting with multiple ^18^F-FDG-avid foci representing metastatic disease are considered unsuitable for surgery, and are treated palliatively with chemotherapy. In the PET-PANC study (one of the larger studies of its kind, which prospectively evaluated the benefits of PET/CT imaging in 589 patients with PDAC across multiple hospitals in the UK), the use of ^18^F-FDG PET/CT influenced management in 45% of patients and led to the avoidance of unnecessary surgery in 20% of patients [10]. Unfortunately, the occurrence of pancreatitis may complicate the interpretation of ^18^F-FDG PET images as this inflammatory response, which coincidentally plays a significant role in PDAC tumorigenesis [11], can also lead to ^18^F-FDG-avid foci. This effect is especially strong in the case of groove, autoimmune, and focal pancreatitis [12], and complicates the differentiation of PDAC from these more benign conditions. Also, glucose intolerance is a frequent complication in patients with PDAC, and high glucose levels can decrease ^18^F-FDG uptake in the tumour. Preclinical and preliminary clinical data further suggest that ^18^F-FDG PET cannot reliably be used for the evaluation of therapy response following chemotherapy or chemoradiotherapy [13, 14].

### ^18^F-FLT

**Fig. 2:**
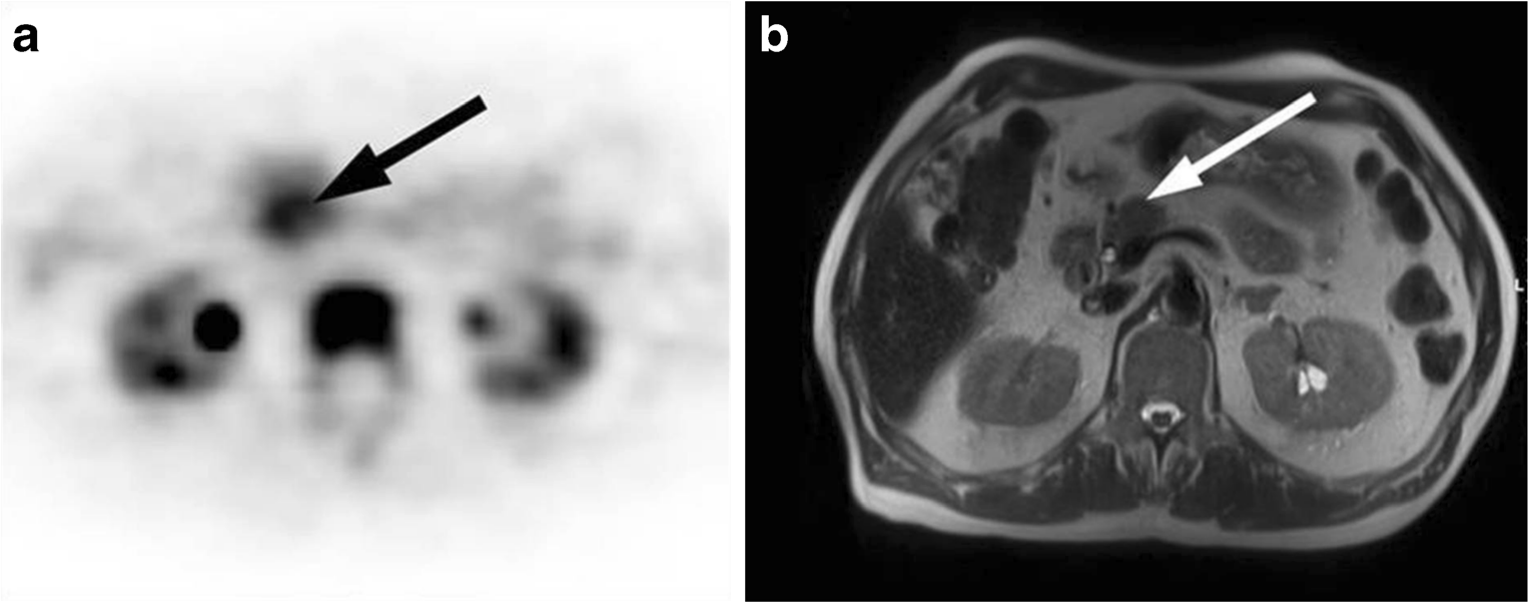
^18^F-FLT PET image in a 70-year-old patient with a 2-cm tumour in the pancreatic head [19]

The ability of [^18^F]-3′-fluoro-3′-deoxy-l-thymidine (^18^F-FLT), an imaging agent designed to measure cell proliferation, to image PDAC has also been evaluated. ^18^F-FLT is an analogue of the nucleotide thymidine, which is generally taken up in highly proliferative tissues [15]. ^18^F-FLT has been evaluated as an early predictor of disease progression in patients with advanced and metastatic pancreatic cancer, but assessment of especially liver metastases may be hampered by high background hepatic activity due to ^18^F-FLT glucuronidation [16, 17]. Early data from Quon et al. indicate that generally low ^18^F-FLT PET tumour uptake results in poor lesion detectability [18], suggesting that ^18^F-FLT is not a good alternative to FDG for PET imaging. A recent study by Wieder et al. looked at the ability of ^18^F-FLT PET imaging to identify those patients with known tumours who might fare worse [19]. In a small cohort of 27 patients, they found that ^18^F-FLT uptake in the tumour was correlated significantly with survival (hazard ratio 1.298, 95% CI 1.001–1.685; *p* < 0.05). An example of a patient with high ^18^F-FLT uptake in the tumour is shown in Fig. 2. They concluded that, although ^18^F-FLT PET is a poor diagnostic imaging method, it may allow risk stratification in patients with resectable pancreatic cancer prior to surgery.

### Hypoxia

PDAC is generally believed to be unusually hypoxic, which contributes to the poor response to chemotherapy and radiotherapy [20]. Although the exact causes of this hypoxic phenotype are not entirely understood, it is known that PDAC is a particularly poorly vascularized tumour. Additionally, the exaggerated desmoplastic response in PDAC is thought to contribute to the hypoxic state, and in turn, to hypoxia-enhancing desmoplasia. The term ‘desmoplasia’ denotes the extensive fibroblastic cell proliferation surrounding the PDAC cell glands, containing extracellular matrix proteins, myofibroblastic pancreatic stellate cells and immune cells. Together, they modulate PDAC growth by providing a scaffold for the cancer cells to grow, as well as growth factors and immune modulators [21]. The mere denseness of the dense desmoplastic reaction prevents oxygen diffusion, but signalling within this microenvironment also contributes to its own advance. For example, pancreatic stellate cells, which make up the bulk of the stroma, under hypoxic conditions stimulate the production of endostatin, an angiostatic factor, by pancreatic cancer cells, thereby reducing angiogenesis and enhancing hypoxia. Also the stroma of PDAC tissue is often hypoxic. These oxygen-lacking tumour areas, at least in mouse models of PDAC, are highly glycolytic and release lactate, which is metabolized by nearby normoxic cancer cells to sustain proliferation. This particularly hypoxic microenvironment is thought to play a significant role in the poor outcome of PDAC patients due to induction of epithelial-to-mesenchymal transition that promotes early metastasis [20].

These considerations provide the rationale for imaging hypoxia using the well-known nitroimidazole-based hypoxia imaging agents, ^18^F-FMISO and ^18^F-FAZA (^18^F-HX4 has also been used by some groups). Yet hypoxia in PDAC tumours is highly heterogeneous, and hypoxia imaging in PDAC is hindered by a propensity to reach diffusive equilibrium only slowly [22, 23]. ^18^F-FMISO uptake in proven PDAC has been correlated with a worse prognosis [24], although ^18^F-FMISO uptake in PDAC is rather limited, with tumour-to-blood ratios of 1.2:1 generally considered as indicative of hypoxia. The same is true for ^18^F-FAZA [23]. ^18^F-HX4 may lead to higher ratios, with ratios between 1.3 and 2.1 reported [25]. Certainly, more research is needed to define the exact role of hypoxia imaging in the management of patients with PDAC.

### Antibodies

Radiolabelled antibody-based imaging of pancreatic tumours in patients was reported as early as 20 years ago in a study in which mucins were targeted using a murine anti-Nd2 antibody [26]. The authors showed a good correlation between pancreatic uptake, measured by planar scintigraphy, and immunostaining of excised pancreatic tumorous tissue by Nd2. However, there was little correlation with CEA or CA19.9 plasma levels. They also concluded that Nd2 imaging could differentiate exocrine pancreatic tumours from benign lesions and PDAC. However, since then, hardly any clinical follow up studies have been performed [27]. One of the few studies investigated the use of ^111^In-labelled amatuximab in mesothelin-expressing cancers [28]. Although antibodies and their fragments hold great potential as the basis for PDAC-imaging agents, given their unrivalled selectivity and affinity, the main challenges in clinical translation of antibody-based imaging continue to be: (1) the cost of producing antibodies in reliable quantities to the required quality standard, (2) the presence of the enhanced perfusion and retention (EPR) effect that causes nonselective extravasation and uptake in tumours with hyperfenestrated vasculature, (3) trapping of antibodies by the reticuloendothelial system that leads to hepatic and splenic uptake, complicating differentiation of primary PDAC and hepatic metastases from physiological uptake, and (4) the lack of chemistry that would allow fast, site-selective and thermodynamically stable radiolabelling of the antibody vector [29, 30]. However, the advent of immunotherapy and its increased deployment in the clinic hails a new start for immuno-PET imaging in which there is increased interest and development.

## Preclinical molecular imaging of pancreatic cancer

Despite there being several imaging agents under evaluation for the management of patients with PDAC (see above), there is most certainly a need for more and better agents. For example, several epitopes and signalling pathways have been under-explored so far, even preclinically, as molecular imaging targets. Hence, molecular imaging of PDAC merits preclinical exploration, including exploration of targets for the tumour stroma [31], cell–cell interactions [32], and PDAC’s inflammatory microenvironment [33]. There is still much room for expansion of metabolic imaging agents for use in PET – and SPECT – as agents targeting aberrant PDAC cell signalling (reviews include [34–36]). The most studied signalling pathway in PDAC is without doubt the RAS-RAF-MAPK pathway, given the near universal mutation of KRAS in PDAC, yet molecular imaging of this crucial signalling axis has so far proven elusive. Other pathways of interest include p53 and SMAD4 signalling, both often mutated in PADC, as well as notch, IGF and WNT signalling [36–38]. Imaging of PDAC, by contrast, focuses mostly on the extracellular changes that arise during PDAC development. Here, we give an overview of some of the radiolabelled imaging agents that have already been evaluated.

### GRP78

GRP78 is a 78-kDa glucose-regulated protein that is known to control structural maturation of glycoproteins. However, it is also expressed on the cell surface where it acts as a receptor for a wide variety of ligands and as an autoantigen [39]. GRP78 has also been described as a receptor of angiogenic peptides and is known to interact with major histocompatibility complex class I. Cell-surface GRP78 expression has been detected in many different cancers, such as breast, liver, prostate, and pancreatic tumour tissue, and has been associated with the development of drug resistance and cell transformation [40, 41].

**Fig. 3:**
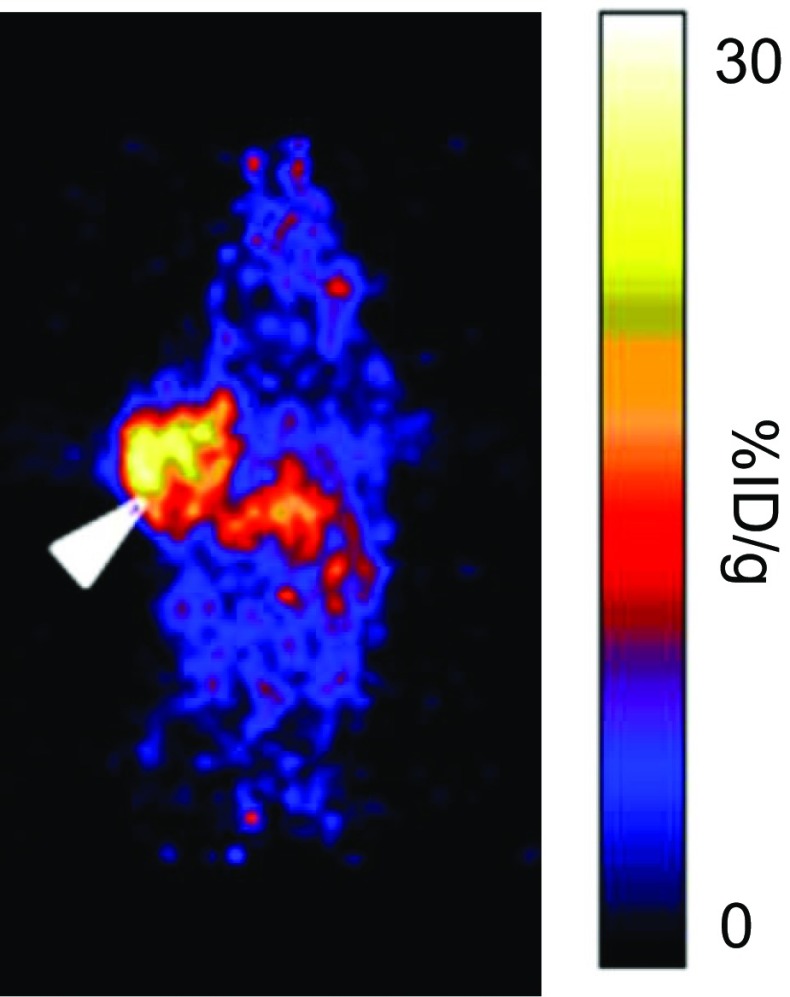
Coronal PET image of a mouse bearing a GRP78-positive BxPC-3 tumour xenograft 48 h after injection of the ^64^Cu-labelled anti-GRP78 antibody MAb159 [44]

The use of anti-GRP78 antibodies in anticancer therapy is being explored given their ability to suppress a wide variety of xenograft tumours by inhibiting PI3K/AKT signalling and by inducing apoptosis [42]. Wang et al. described a ^64^Cu-labelled anti-GRP78 monoclonal antibody, MAb159, which binds to GRP78 with an affinity in the low nanomolar range (*K*_d_ = 1.7 nM) [43, 44] Although only modest tumour uptake was observed in GRP78-overexpressing BxPC-3 xenografts in mice (18 ± 1.0%ID/g at 48 h after injection; Fig. 3), no uptake above EPR was observed for a ^64^Cu-labelled nonspecific isotype control antibody, suggesting that labelled antibodies such as this one may have translational potential.

### Transferrin

The transferrin receptor 1 (TfR1, or CD71) is significantly involved in iron homeostasis and proliferation [45]. This cell membrane receptor binds to iron-loaded transferrin to import iron into the cell. Upon endocytosis, iron is released and the receptor–transferrin complex is recycled back to the cell surface. The transferrin receptor is a marker of a malignant phenotype in PDAC [46], its expression correlates with a worse prognosis. It is also thought to modulate mitochondrial respiration and generation of radical oxygen species [47]. The advantage of transferrin receptor imaging is that it may also have applications in the detection of pancreatic neuroendocrine and lung cancer.

Holland et al. used transferrin itself to target the transferrin receptor in a glioblastoma xenograft mouse model [48, 49]. They found tumour uptake of >10%ID/g at 24 and 48 h after administration of ^89^Zr-transferrin, also labelled using the iron-binding siderophore desferrioxamine (DFO) as a metal ion chelator rather than relying on the transferrin itself to bind to the ^89^Zr^4+^ ion. This compound may therefore also hold promise for PDAC imaging.

**Fig. 4:**
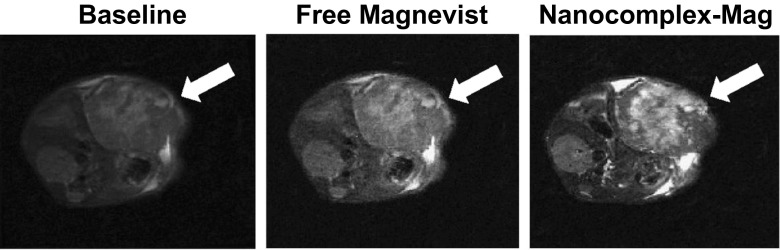
MRI imaging in an orthotopic mouse model of pancreatic cancer 4 months after surgical implantation of the tumour showing the difference in signal between intravenously administered conventional contrast agent (free Magnevist) and the TfRscFv-Lip-Mag complex [50]

Pirollo et al. generated what they called a ‘nanodelivery platform’ for MRI imaging of transferrin comprising an antitransferrin receptor single-chain antibody fragment linked to a liposomal nanoparticle containing Gd-DTPA (Fig. 4) [50]. They found increased uptake in PDAC xenografts in mice, and improved lesion delineation, but unfortunately did not quantify their results. Sugyo et al. used a ^89^Zr-labelled anti-transferrin receptor monoclonal antibody (TSP-A01) for PET imaging in MiaPaCa-2 xenograft-bearing mice as a precursor for antitransferrin receptor immunotherapy [51]. Tumour uptake was relatively modest (for an antibody) at 12 ± 2.3%ID/g at 48 h after intravenous injection but provided the necessary contrast for lesion visualization.

### Mesothelin

Mesothelin is a 40-kDa transmembrane protein that is normally expressed in mesothelial cells where it activates STAT, AKT and MAPK signalling pathways [52]. Under physiological conditions, mesothelin is expressed at relatively low levels, but is overexpressed in virtually all malignant mesotheliomas as well as in pancreatic adenocarcinomas. One study even found mesothelin expressed in 60 out of 60 human PDAC samples [53], and the extent of overexpression proved a strong predictor of a worse prognosis. Importantly, mesothelin is not detected in normal, healthy pancreas [52]. It is therefore no surprise that mesothelin is being investigated as a target for immunotherapy in ongoing clinical trials evaluating among other approaches antibody–drug conjugates including anetumab ravtansine [54].

**Fig. 5:**
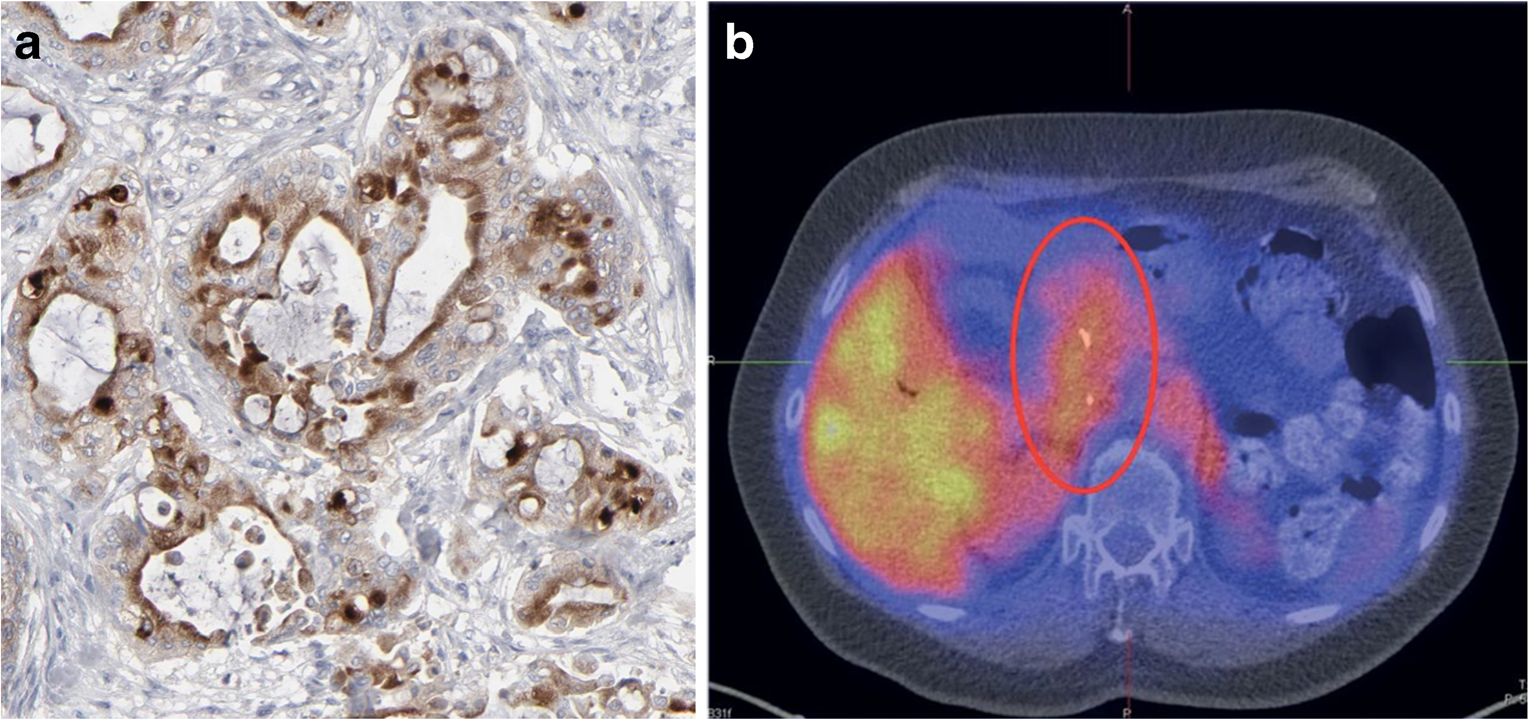
**a** Mesothelin in PDAC (protein atlas: 11/12 positive [56]). **b** ^89^Zr-MMOT0530 PET/CT image in a patient with PDAC shows the primary tumour (*red oval*), as well as uptake in healthy liver [55]

Lamberts et al. used an antimesothelin antibody for PET imaging of PDAC to prove delivery of the antibody to tumour tissue. Using the anti-mesothelin antibody AMA radiolabelled with ^89^Zr using a DFO chelator, they found uptake in HPAC and CAPAN-2 xenografts of up to 12%ID/g 6 days after injection. The authors went on to use a similar construct in patients with ovarian cancer or PDAC before treatment with a mesothelin-targeting antibody–drug conjugate (Fig. 5) [55]. The mean SUV in PDAC tumours was 11.5, although some lesions showed SUVs as high as 20 and as low as 5, with relatively high uptake in normal tissue uptake found, as expected, (mean SUV_max_ approximately 14 in the liver).

### Integrins: α_v_β_6_, α_v_β_3_

Integrin expression has been targeted since the late 1990s for molecular imaging using peptides containing the tripeptide RGD sequence which binds preferentially to integrins α_v_β_3_, α_v_β_5_ and α_v_β_6_ [57], an interaction that had been known about for a decade previously. For an excellent review of this particular topic, see reference [58]. A wide variety of MRI, optical and radiolabelled imaging agents have been developed to target integrins over the years, mostly focusing on α_v_β_3_ in the context of tumour neovascularization [59]. The latter can be targeted by RGD peptide-based imaging agents, although this is not specific for the α_v_β_6_ overexpressed in PDAC [60], and that has been pursued for therapeutic benefit in that setting [61]. The use of antibodies and peptides against α_v_β_6_ for PDAC therapy is being investigated, with promising results reported with the combination of antibody therapy and gemcitabine in a mouse model [62].

**Fig. 6:**
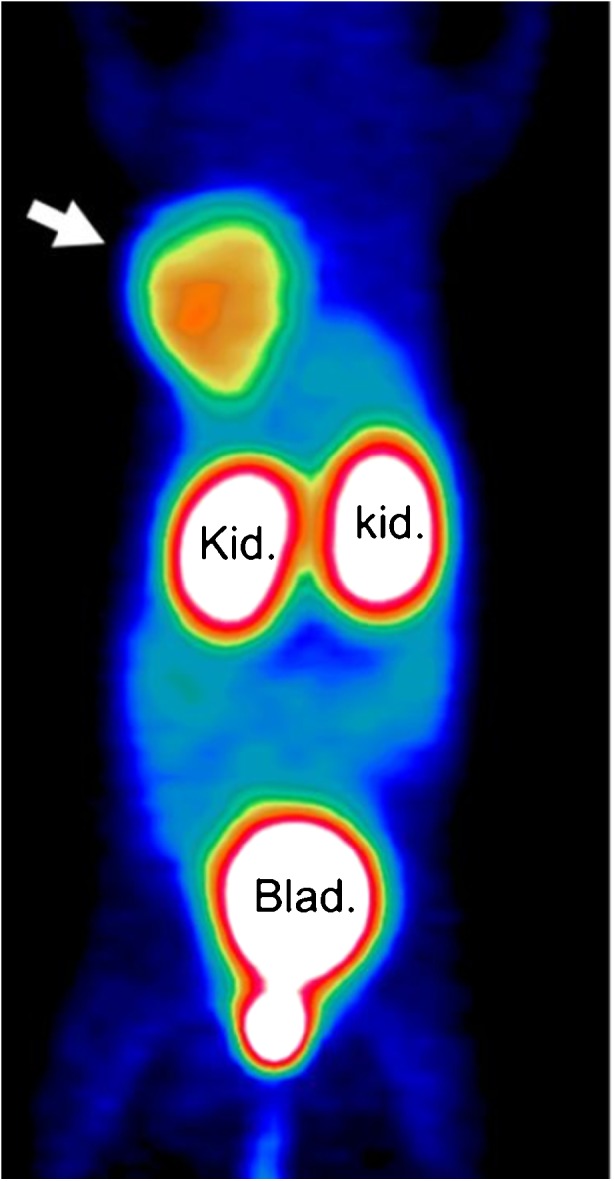
^68^Ga-avebehexin PET image (maximum-intensity projection, 60 min after injection) of a H2009-bearing SCID mouse. The tumour is indicated by the *arrow*. *Blad.* bladder, *Kid.* kidneys [63]

One α_v_β_6_-targeting compound, ^68^Ga-avebehexin, comprises a triazacyclononane-triphosphinate (TRAP) chelator for ^68^Ga labelling, linked to three α_v_β_6_ integrin-selective cyclic nonapeptides [63]. This conjugate was specifically taken up by α_v_β_6_-expressing H2009 lung cancer cells, with uptake of around 0.65%ID/g, with some specific uptake in the stomach (0.52%ID/g) and intestines, but negligible uptake in healthy murine pancreas (0.07%ID/g; Fig. 6). However, similar contrast was obtained targeting α_v_β_3_ using ^68^Ga-NODAGA-RGD in a genetically engineered spontaneous mouse model of PDAC, the KPC model [60, 64]. Whether α_v_β_6_ or α_v_β_3_ is the optimal target for PDAC imaging remains to be determined.

### Tissue factor

Overexpression of tissue factor (TF, CD142) has been associated with increased tumour growth, tumour angiogenesis, and metastatic potential in many malignancies, including pancreatic cancer, and soluble TF may also contribute to activation of the coagulation system in pancreatic cancer [65]. Its physiological functions include the processing of prothrombin to thrombin, an essential factor in blood clotting. Hernandez et al. synthesized a ^89^Zr-labelled anti-TF monoclonal antibody, ALT-836. They observed very good uptake in BxPC-3 TF-overexpressing tumours (32 ± 6.0 %ID/g) in contrast to 2.3 ± 0.5%ID/g in tumours in which specific uptake was blocked by an excess of cold, unlabelled antibody [66]. The uptake of ALT-836 was much higher than that of an earlier ^64^Cu-labelled version (15%ID/g) [67]. Takashima et al. used another anti-TF antibody (clone 1849) labelled with ^111^In to image orthotopic gliomas, with similar results [68].

### Neurotensin receptors

Neurotensin is a 13 amino acid peptide first isolated in 1973 from bovine hypothalamus [69]. It is normally present in the gastrointestinal tract and the brain, where it is thought to trigger a wide variety of central and peripheral functions through its interaction with three neurotensin (NTS, or NT) receptors: NTSR1, NTSR2, and NTSR3. NTSR1 is a G protein-coupled transmembrane protein whose functions include blood pressure, blood sugar and temperature homeostasis. NTSR1 is known to be overexpressed in PDAC primary and metastatic tumour masses, as well as in high-grade PanINs [70], and also in prostate and colorectal cancer. For this reason, it too has been targeted for molecular imaging with radiopharmaceuticals for quite some time.

**Fig. 7:**
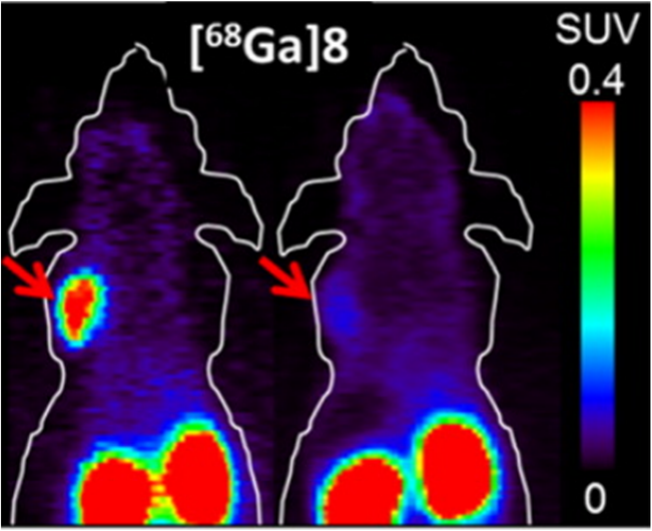
Coronal small-animal PET images of HT29 tumour-bearing immunodeficient mice injected with ^68^Ga-8, a radiolabelled neurotensin peptide analogue. The mouse on the right received a blocking dose of cold, unlabelled compound to saturate the receptor [71]

Imaging of NTSR1 (also sometimes called NTR) has often been based on NTS, the natural ligand. The main challenge here is that the C-terminal NTSR1 binding domain NTS(8–13) is rapidly degraded in vivo by endogenous peptidases. Therefore, efforts have focused on the introduction of non-natural amino acids or variation of the amino bonds to prevent degradation while preserving affinity of the molecule for NTSR1 [71]. One successful example from amongst a multitude of studies is a systematic study of the NTSR1 and NTSR2 binding affinity of a dozen compounds labelled with ^18^F or ^68^Ga. One of these peptides showed acceptable NTSR1 selectivity (fourfold higher affinity for NTSR1 than for NTSR2). Tumour uptake and pharmacokinetics were evaluated in vivo (Fig. 7), and the tracer showed uptake of up to 1.6 ± 0.35%ID/g in HT29 colorectal adenocarcinoma tumours, providing excellent contrast with respect to normal tissue (tumour-to-blood ratio 31, tumour-to-muscle ratio 3.2) [71].

Another set of NTSR1 imaging agents is based on small-molecule NTSR antagonists, such as SR142948A. One example was labelled though a Cu-assisted click reaction with ^18^F-2-deoxy-2-fluoroglucosyl azide [72]. The authors showed good receptor affinity of around 0.98 nM (*K*_d_), and HT29 tumour xenograft uptake in mice of up to 0.7%ID/g, with much lower uptake in most normal tissues, at 1 h after injection. Unfortunately, intestinal uptake was high, possibly due to hepatobiliary excretion, potentially limiting applications for the imaging of stomach and pancreatic tumours.

## Cathepsins

**Fig. 8:**
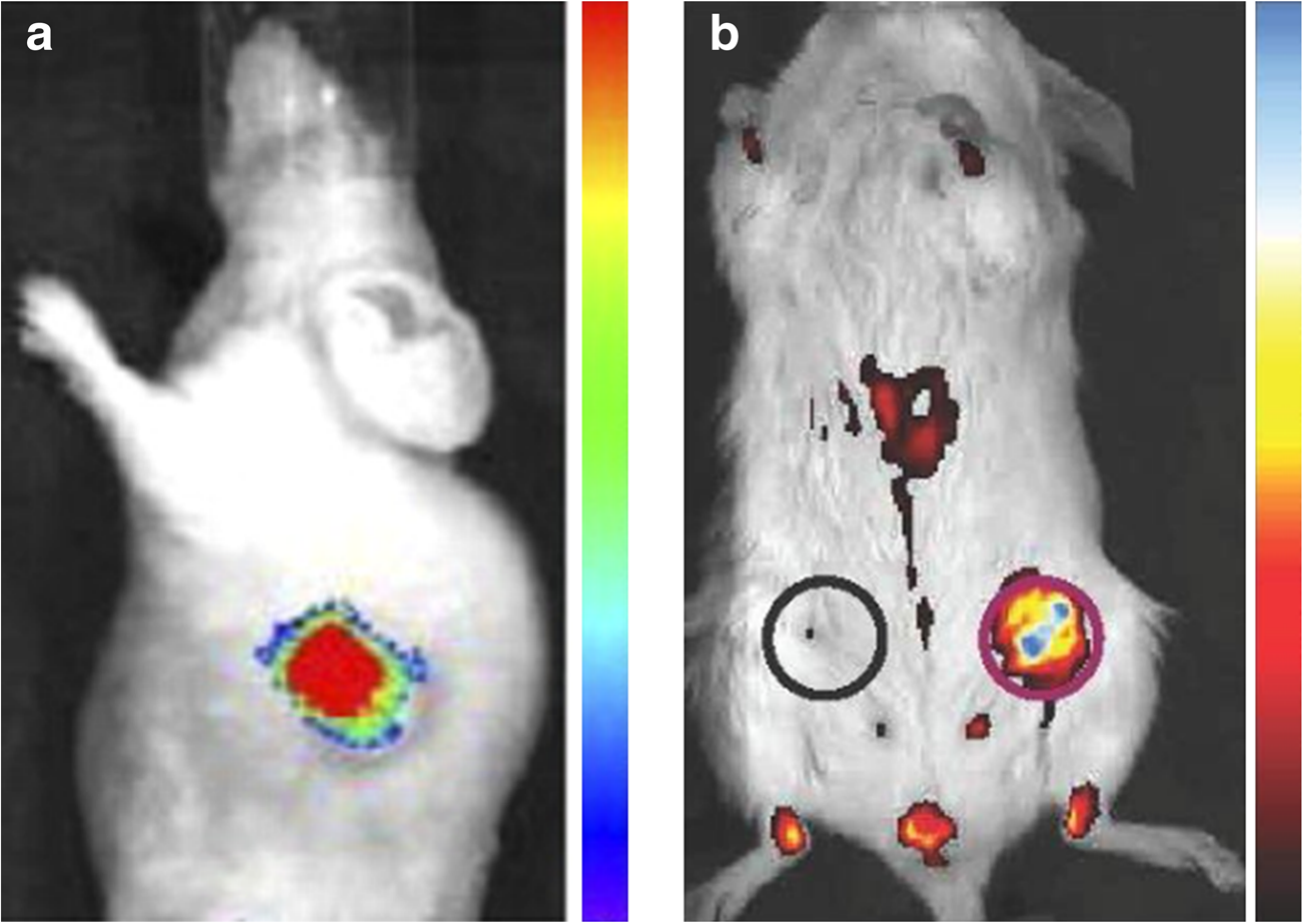
**a** Representative in-vivo image of a human primary pancreatic cancer tumour xenograft in a mouse after administration of a Cy5 fluorophore-labelled cathepsin E substrate [74]. **b** A Cy5.5-labelled cathepsin B-targeting DARPin is taken up in a 4T1 allograft murine breast tumour (*red circle*), but not in a healthy mammary fat pad (*black circle*) [75]

In the pancreas, cathepsin E is not expressed in normal healthy tissue, but is present in nearly all PDAC tissue. Cathepsins are a family of proteases implicated in the regulation of angiogenesis and invasion during cancer progression, and are highly upregulated in pancreatic cancer, contributing to the development and progression of the cancer phenotype [73]. A Cy5 fluorophore-labelled cathepsin E substrate was developed as an imaging probe by Cruz-Monserrate et al. [74]. They showed uptake of the labelled substrate in MDA PATC-3 murine PDAC cells, as well as PDAC tumours in KPC mice, with PanIN precursor lesions showing slightly lower uptake (Fig. 8).

In addition, the role of cathepsin B as a driver of tumour progression in pancreatic cancer has been demonstrated [76]. This was exploited by Kramer et al. who developed a cathepsin B-binding DARPin, an ankyrin repeat protein, with very high affinity (*K*_d_ = 35 pM). When tagged with the fluorophore Cy5.5, this DARPin (8h6) was taken up in cathepsin B-positive 4T1 subcutaneous allografts [75]. The lack of radionuclide-labelled imaging agents makes direct comparison with the other compounds discussed in this overview challenging.

### CEA

**Fig. 9:**
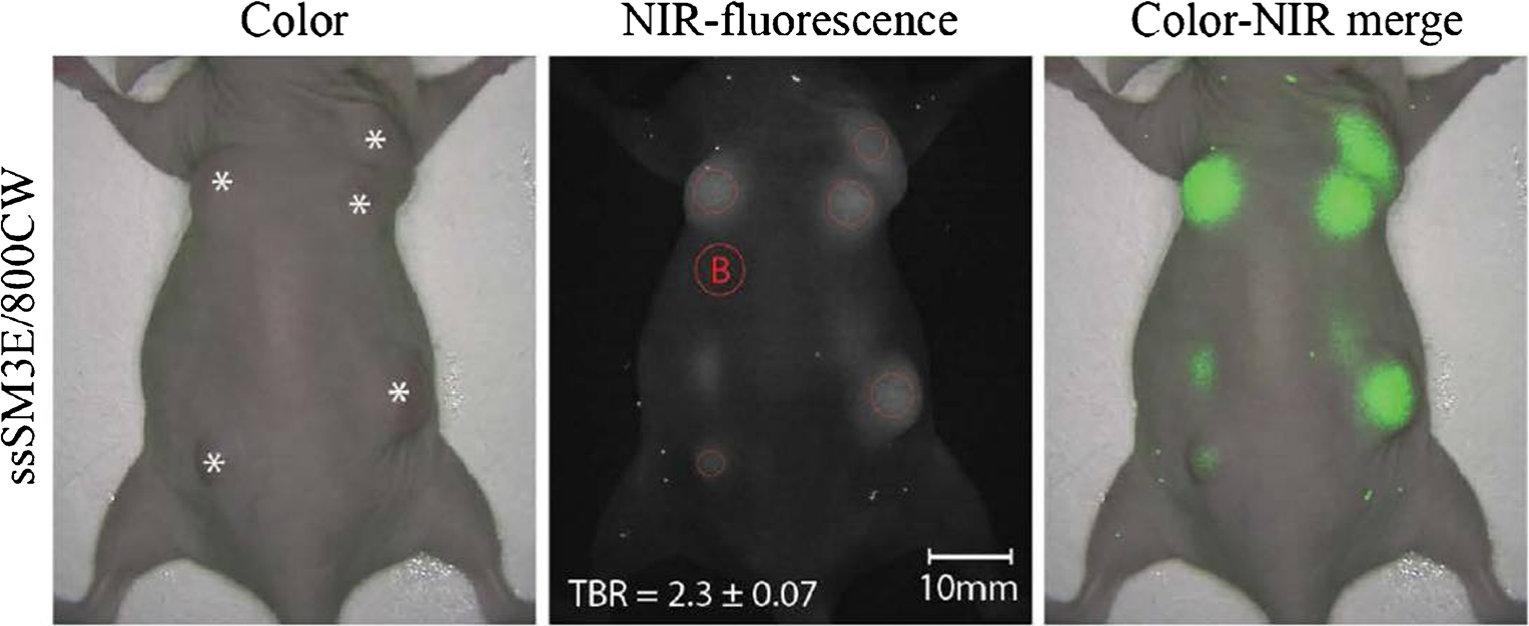
NIR fluorescence imaging in a mouse bearing subcutaneous PDAC tumours acquired 72 h after injection of CEA-targeting ssSM3E/800CW or F73/800CW [78]

Serum expression of carcinoembryonic antigen (CEA, CD66e, CEACAM5) has been known for some time to be a prognostic biomarker in PDAC, albeit with rather poor sensitivity and specificity [77]. Boonstra et al. used a single chain antibody fragment (scFv) that binds to CEA and labelled it with the near-infrared fluorescent dye 800CW [78], and evaluated it in a mouse model of colorectal cancer (Fig. 9). High tumour-to-background ratios were found 72 h after intravenous administration in mice bearing subcutaneous xenografts. Uncharacteristically for a single chain fragment, pharmacokinetics were rather slow, and liver uptake was high relative to other organs. In another study by the same authors, a correlation was found between serum CEA levels and PDAC tumour expression of CEA, allowing selection of PDAC patients who might benefit from CEA-targeted imaging [79].

**Fig. 10:**
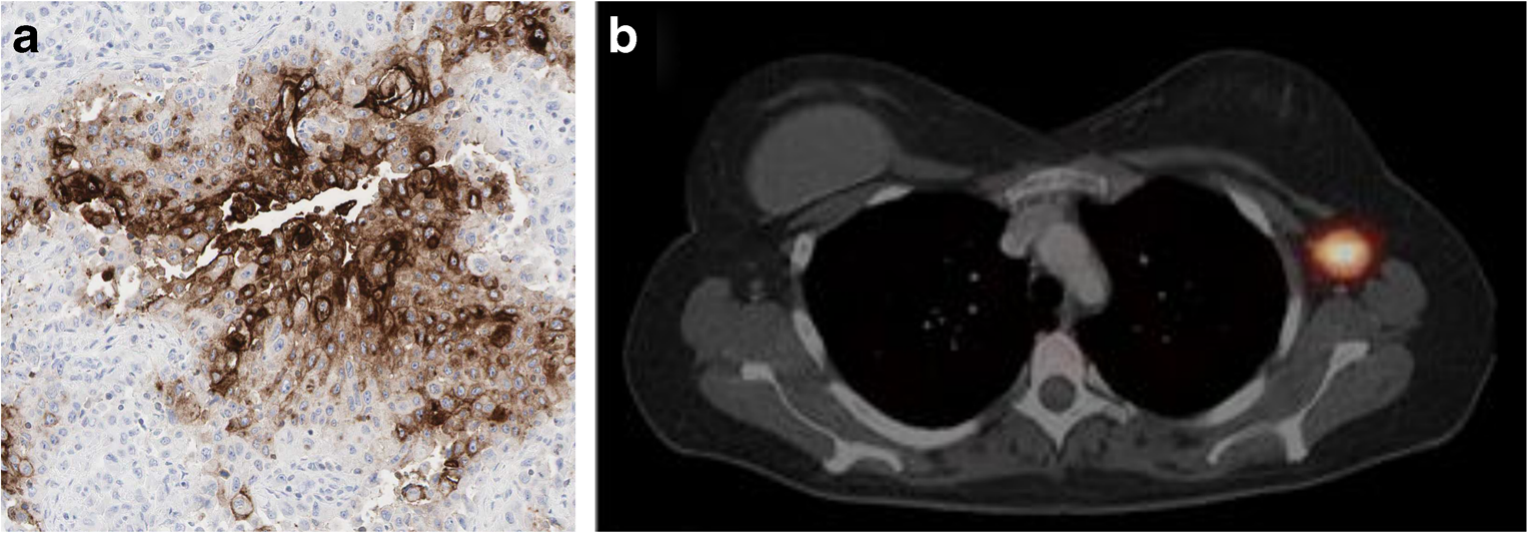
**a** CEA expression in human PDAC tissue (protein atlas [56]) **b** SPECT/CT image in a 38-year-old patient acquired 24 h after injection of ^111^In-IMP288 (185 MBq, 25 μg) pretargeted with 75 mg of TF2 (1-day interval) [80]

A bispecific engineered antibody for pretargeted imaging of CEA in combination with a radiolabelled hapten (^111^In-IMP288) has been evaluated in a series of studies up to a first-in-man study [80]. This followed earlier in vitro and preclinical studies by Rossi et al. [81], Goldenberg et al. [82] and Schoffelen et al. [83]. They showed good targeting of CEA-expressing lymph node metastases in patients with colorectal cancer (Fig. 10), suggesting that pretargeting may be an excellent way to circumvent the unfavourable liver, spleen and intestinal uptake of other molecular imaging agents. PDAC imaging has yet to be explored using this system.

### CA19.9

**Fig. 11:**
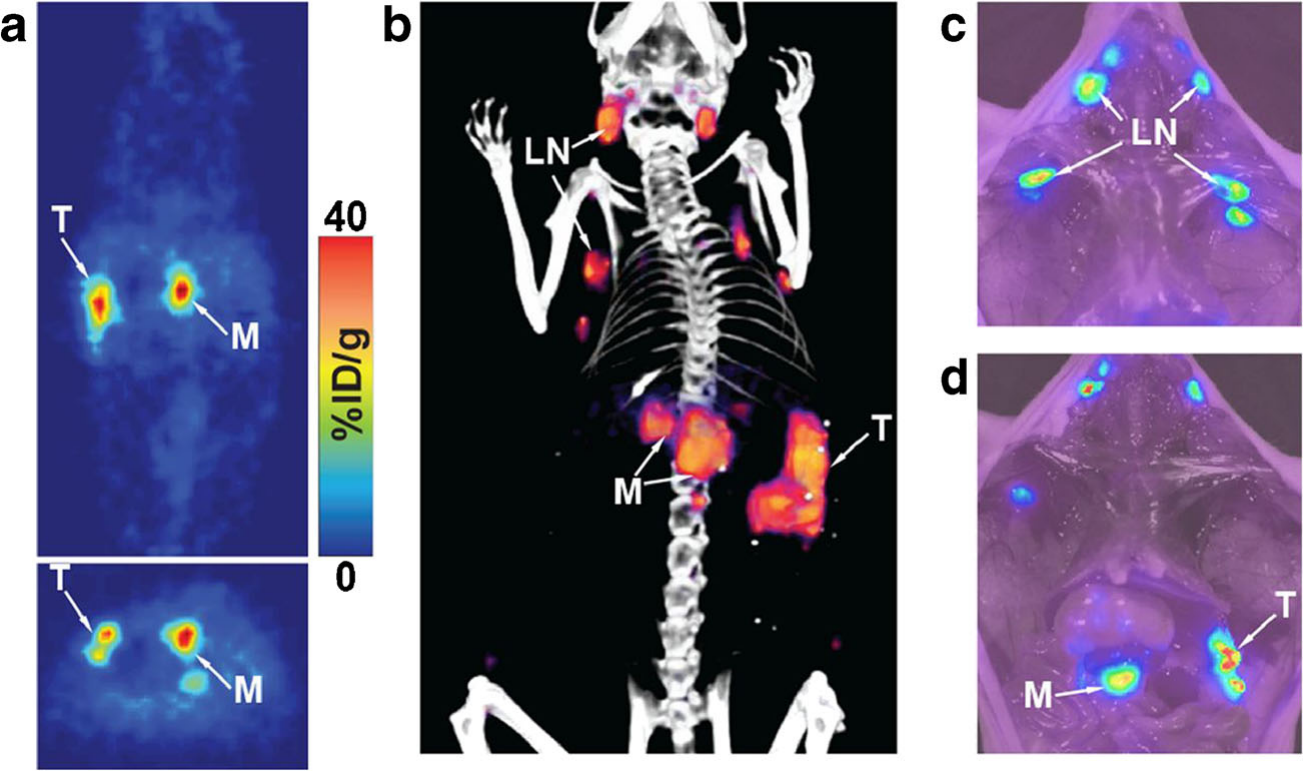
PET, PET/CT, and near infrared imaging in an orthotopic PDAC mouse model using a ^89^Zr-labelled anti-CA19.9 antibody (*LN* lymph node, *M* metastasis, *T* tumour) [84]

Another innovative and promising area of work is that on imaging CA19.9 in the local tumour environment. CA19.9 levels in blood serum are used clinically as a PDAC biomarker, but with limited sensitivity. However, CA19.9 originates in the PDAC tissue itself, and local concentrations within the tumour are therefore many times higher than circulating levels. Houghton et al. therefore tagged the fully human monoclonal anti-CA19.9 antibody 5B1 in a site-specific manner with DFO for ^89^Zr labelling, or with a near-infrared dye [84, 85]. Even though the modified antibodies showed a relatively poor affinity for their target (*K*_d_ = 51 nM), they showed excellent uptake in CA19.9-positive BxPC-3, yet negligible uptake in negative control tumours not expressing CA19.9 (tumour uptake up to 102 ± 26%ID/g in subcutaneous BxPC3 tumour xenografts, with tumour-to-blood ratios as high as 20:1), and good uptake in orthotopic PDAC xenografts (40%ID/g; Fig. 11). The radiolabelled construct is now under evaluation in clinical trials [57].

### Imaging drug delivery, drug efficacy: gemcitabine delivery/resistance

Most of the work discussed above focused on the development of diagnostic or prognostic molecular imaging agents, or looked at the specificity of potentially therapeutic antibodies. However, another major challenge in PDAC patients is the lack of response to the chemotherapy agents that are currently employed to kill the tumour cells, and remain standard clinical practice. To tackle this challenge, several groups have looked at using molecular imaging to monitor a drug’s effects, or alternatively have sought to radiolabel the drugs themselves, and so visualize their delivery, or indeed the absence thereof. Below we briefly describe some recent work on imaging DNA damage and on the delivery of gemcitabine.

### γH2AX

**Fig. 12:**
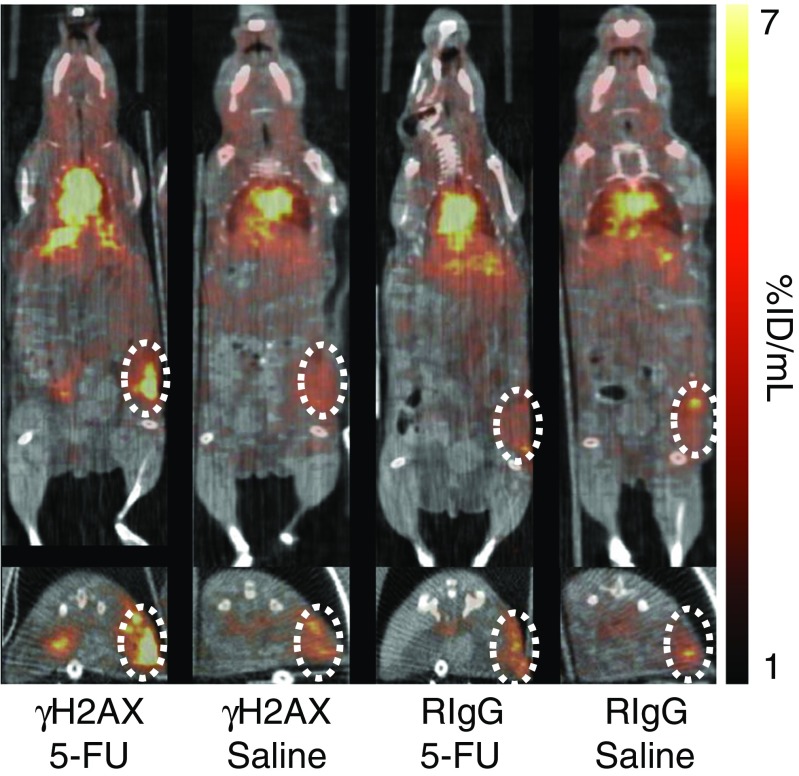
Monitoring 5-FU therapy with ^89^Zr-anti-γH2AX-TAT: PET/CT images show coronal (*top*) and transaxial (*bottom*) sections intersecting the centre of the allograft tumour (*dotted circles*) [13]. Tumour uptake of a nonspecific control antibody (RIgG) was not different between 5-FU-treated and vehicle-treated animals

Knight et al. found that targeting of the DNA damage marker, γH2AX, predicted the response to chemotherapy using 5-FU, gemcitabine or capecitabine [13]. These authors demonstrated that uptake of a ^89^Zr-labelled anti-γH2AX antibody modified with a cell-penetrating peptide, TAT, was significantly higher in subcutaneous PDAC allograft tumours of mice that had received chemotherapy than in vehicle-treated mice. ^18^F-FDG, on the other hand, did not provide a useful indication of therapeutic response (Fig. 12).

### Radiolabelled nucleotide analogues

Chemotherapy agents used for the treatment of PDAC included nucleotide analogues, such as 5-FU, gemcitabine and capecitabine. The inability of these compounds to accumulate in PDAC tissue due to the intense desmoplastic reaction has led some groups to develop radiolabelled analogues of these drugs to study tumour delivery. This is especially relevant given the numerous clinical trials attempting to target the stromal tissue density in PDAC for therapeutic gain [86]. Liang et al. [87] developed a radiolabelled gemcitabine analogue, ^18^F-FAC, and also a deoxycytidine kinase (DCK) substrate. They demonstrated that ^18^F-FAC accumulates selectively in DCK-positive tumour xenografts. Furthermore, ^18^F-FAC PET imaging predicted the response to gemcitabine. Russell et al. showed that ^18^F-l-FAC uptake in orthotopically grown PDAC xenografts correlated well with the delivery of a ^14^C-labelled isotopologue of gemcitabine [88]. The high uptake of ^18^F-FAC in the intestinal lining and in inflamed tissue [90] may, however, be a disadvantage for PDAC imaging, where pancreatitis is ever present, yet certainly provides a tool for assessing drug delivery.

## Challenges

Despite these recent advances, a great number of challenges remain in the molecular imaging of PDAC. In general, there is a well-recognized lack of validated biomarker epitopes to use as targets for imaging agents (or imaging biomarkers). A recent EORTC panel review comprehensively highlighted these as the need for: technical assay validation and GMP production of the imaging agent; biological and clinical validation in multiple model systems and in multiple centres; assessment of cost effectiveness; standardization across centres including accreditation systems; and, finally, the need for ongoing re-evaluation of the precision of an imaging biomarker [90]. Not all of the imaging targets discussed above have been stringently evaluated against these criteria. Most pressingly, imaging targets should be validated using patient samples, for example using immunohistochemistry. For PDAC, limited access to these samples is often a major obstacle. Given the need for large, multicentre trials to finally validate imaging agents for PDAC, the cost and therefore the feasibility of these studies become a limiting factors.

The wide variety of PDAC imaging agents that have been explored preclinically exemplifies the lack of one clear biomarker or target for PDAC imaging. Direct comparison between imaging agents is severely complicated by: (1) the lack of quantitative information regarding relative (over)expression of the various epitopes for imaging in PDAC versus normal pancreatic tissue, (2) the lack of quantitative data regarding the affinity of some of the imaging agents, and (3) the variety of preclinical models that have been used to evaluate these imaging agents. The eventual choice of imaging agent will be informed by a combination of the relevance of the target epitope, tumour-to-background contrast, and tumour uptake.

Another challenge is the use of a suitable animal model to predict the usefulness of an imaging agent for the human disease. Subcutaneous xenograft models are a far cry from the complexity of PDAC. They provide an excellent platform for providing proof-of-principle of an imaging agent, but may offer unrealistic levels of target epitope expression relative to clinical presentation, a measure that is seldom quantified. On the other hand, orthotopic implantation of PDAC cancer cells seems underused in molecular imaging, although this model does show some of the complications such as desmoplastic responses seen in the clinic. Only a few genetically engineered animal models of pancreatic cancer that replicate the human disease exist [91], and of these, the KPC mouse model is by far the most used, and is the only one to have been employed in nuclear medicine imaging [88, 92, 93]. However, access to these often complex and expensive models is a key limiting factor [94]. To accurately compare different developments, there is a need for standardization of the animal models used. Additionally, imaging in patient-derived xenograft models (PDX) may provide many benefits for evaluating the response to therapy in an individual patient [94], but requires a logistically intricate setup.

The existence of desmoplasia, the build-up of stiff tissue with overproduction of extracellular matrix proteins around the tumour site, is a challenge for PDAC imaging both in the clinic and in preclinical mouse models. It is believed that desmoplasia can severely limit blood supply, and it has been suggested as a major factor in limiting the delivery of blood-borne imaging agents, as well as drugs, to the tumour site. The various preclinical PDAC tumour models also show different levels of desmoplasia, further complicating direct comparison between imaging agents and models. Desmoplasia is least evident in subcutaneous xenografts, highlighting the care that should be taken in extrapolating preclinical results to the clinic. Interestingly, this desmoplasia does not seem to be an issue for even a large antibody-based CA19.9 imaging probe, either in orthotopic xenografts or in patients with PDAC [57, 84, 85]. These seemingly opposing findings indicate that more insight into the relationship of PDAC vascularity and delivery of imaging agents is required.

Since the pancreas is a deep-seated organ in close proximity to the liver and intestines and because metastases occur in the liver, there is a need for imaging agents with a ‘clean’ elimination profile, preferably with a renal excretion pattern. To meet these pharmacokinetic requirements, renally cleared small molecules, peptides and nanobodies, as well as pretargeting techniques, need to be developed rather than using directly radiolabelled antibodies. The latter show significant liver uptake and may hinder the detection of ubiquitous liver metastases.

Finally, the cost of nuclear medicine procedures in the management of patients with pancreatic cancer should be addressed. The cost of such a procedure must be weighed against the potential benefits, e.g. extending life expectancy or enhancing quality of life. For early detection of PDAC, molecular imaging could close the diagnostic sensitivity gap between small, undetectable, or even premalignant disease, and lesions visible on CT or MR and could assist in improving the identification of patients who could benefit from radical treatments including radiochemotherapy as well as surgery. Likewise, the benefits of an imaging procedure that allows early detection of a failing therapy (in more than 90% of patients [95]) far outweigh the cost of continuing ineffective therapy, in terms of both the financial cost and its effect on the patient’s quality of life and treatment-related morbidity/mortality.

## Conclusion

In the field of pancreatic cancer research, much despondency during the past 40 years is now giving way to quiet enthusiasm due to new investment and developments in basic research, biomarker discovery and novel therapy combinations. We are strongly of the opinion that nuclear medicine will play a big role in this field. The ‘right’ choice of imaging agent is still a matter of debate, with a wide variety of tools being developed targeting an equally wide range of signalling pathways involved in PDAC. Unfortunately, most novel and potentially game-changing imaging approaches remain untested clinically, or even lack verification of the validity of the imaging target in clinical samples, and much work remains to verify the clinical applicability of imaging agents.
